# Impact of the Lockdown Due to the COVID-19 Pandemic on Patients With Inflammatory Bowel Disease

**DOI:** 10.3389/fmed.2021.649759

**Published:** 2021-12-10

**Authors:** Yu Nishida, Shuhei Hosomi, Koji Fujimoto, Rieko Nakata, Naoko Sugita, Shigehiro Itani, Yuji Nadatani, Shusei Fukunaga, Koji Otani, Fumio Tanaka, Yasuaki Nagami, Koichi Taira, Noriko Kamata, Toshio Watanabe, Satoko Ohfuji, Yasuhiro Fujiwara

**Affiliations:** ^1^Department of Gastroenterology, Osaka City University Graduate School of Medicine, Osaka, Japan; ^2^Department of Public Health, Osaka City University Graduate School of Medicine, Osaka, Japan

**Keywords:** COVID-19, inflammatory bowel disease, lockdown, ulcerative colitis, Crohn's disease

## Abstract

**Background:** The government of Japan declared a state of emergency on April 16, 2020, owing to the coronavirus disease 2019 (COVID-19) pandemic. The subsequent lockdown altered lifestyles and worsened mental illnesses. Inflammatory bowel disease (IBD) is an intestinal disorder that is affected by environmental factors. Therefore, we aimed to assess the effects of COVID-19 and the state of emergency on the lifestyle and disease activity of patients with IBD.

**Methods:** We conducted a questionnaire survey on patients with IBD from June 16 to August 21, 2020 during their regular follow-up at our hospital, 2 months after the state of emergency was declared.

**Results:** Overall, 241 patients with ulcerative colitis (UC) and 210 with Crohn's disease (CD) completed the survey, of which 82 (34%) and 97 (46%) patients, respectively, reported disease exacerbation within 2 months after the lockdown. Multivariate logistic regression analysis identified age at enrollment (odds ratio, OR 0.98, 95% CI 0.96–0.99; *P* < 0.05), sleep hours (OR, 0.74; 95% CI, 0.57–0.97; *P* < 0.05), and increased stress due to the COVID-19 pandemic (OR, 6.06; 95% CI, 1.79–20.50; *P* < 0.01) as independent factors associated with UC exacerbation. Patients with exacerbated CD were younger at CD onset and had higher patient-reported outcome 2 scores before the state of emergency than patients with non-exacerbated CD. On multivariate analysis, age (OR, 0.97; 95% CI, 0.95–0.99; *P* < 0.01) and active disease before the state of emergency (OR, 2.20; 95% CI, 1.23–3.95; *P* < 0.01) were independently associated with CD exacerbation.

**Conclusions:** Improving sleep quality and preventing psychological stress may be crucial in IBD management during a pandemic, especially in young patients.

## Introduction

In December 2019, coronavirus disease 2019 (COVID-19), which is caused by severe acute respiratory syndrome coronavirus 2 (SARS-CoV-2), emerged in Wuhan, China, and the outbreak rapidly spread worldwide (1). It was considered a global health emergency by the World Health Organization. One measure that was adopted by the governments of many countries, especially those more affected by the pandemic, was the lockdown of cities. Consistent with other countries' policies, the government of Japan declared a state of emergency on April 16, 2020, which continued until May 25, 2020. Central and local governments could request or instruct behaviors to prevent the spread of infection, such as school closure, social distancing, and quarantine. Although this approach was partially successful in temporarily preventing the spread (2), concerns were raised regarding the negative impact of these measures not only in terms of economics but also for mental and physical health (3, 4). The lockdown altered sleep, exercise, and nutrition patterns; compromised treatment compliance; increased childcare and work burden (owing to the lack of a workforce); and worsened mental illnesses, such as anxiety and depression (5–8).

Inflammatory bowel diseases (IBDs), comprising ulcerative colitis (UC) and Crohn's disease (CD), are intestinal disorders affected by environmental factors, such as sleep, stress, diet, and smoking (9–13). However, few studies have evaluated the relationship between lockdown measures to control the COVID-19 pandemic and IBD exacerbation. Therefore, this study aimed to assess the effects of the COVID-19 pandemic and state of emergency on the lifestyle and disease activity of patients with IBD.

## Materials and Methods

### Study Design and Participants

This study was conducted through a questionnaire survey among patients with IBD during their regular follow-up at a hospital in Japan, 2 months after the initiation of the state of emergency (from June 16 to August 21, 2020). We asked all patients with IBD who visited the hospital during this period to complete the questionnaire. Patients with repeated visits were investigated only once. The exclusion criteria were a diagnosis of IBD in the last 3 months, inability to complete the questionnaire despite assistance, presence of colostomy or ileostomy, and history of total proctocolectomy with ileal pouch-anal anastomosis.

### Questionnaire Design

The questionnaire included questions regarding the patient's epidemiological history of COVID-19, demographic data (sex, age at recruitment, and age at disease diagnosis), gastrointestinal symptoms, lifestyle (sleeping time, working time, walking time, exercise time, and number of meals) before and after the declaration of the state of emergency, stress related to the state of emergency (due to childcare burden), COVID-19, family budget, inability to exercise, staying indoors, IBD, and worsening of diet and nutritional status), and current medication use.

### Evaluation

Gastrointestinal symptoms were assessed before and after the state of emergency (from April 16 to May 15) using the 6-point Mayo score (14, 15) and patient-reported outcome 2 (PRO2) score (16) for UC and CD, respectively. Severe active UC, moderate active UC, mild active UC, and UC remission were defined as a 6-point Mayo score of ≥ 5, 3–4, 2 and 0–1, respectively (15). Severe active CD, moderate active CD, mild active CD, and CD remission were defined as a PRO2 score of ≥34, 14–33, 8–13, and 0–7, respectively. Patients with mild, moderate, or severe UC were defined as having active disease (16). The primary endpoint was disease exacerbation defined as an increase in the 6-point Mayo or PRO2 scores. “Stress related to the state of emergency” was defined as newly emerging stress during the state of emergency. Deterioration of adherence was defined as an increase in the number of times a patient forgot to take a prescribed medication within a week after the state of emergency.

### Statistical Analysis

Continuous variables are summarized as medians and interquartile ranges. The differences in clinical characteristics were compared using either the chi-square test or Fisher's exact test for categorical variables and the Mann–Whitney U test for continuous variables. Multivariate logistic regression analyses were performed to identify factors associated with exacerbation. Variables in the multivariate analysis were selected among those showing significant differences in a comparison between exacerbated and non-exacerbated patients, and based on known risk factors for exacerbation.

A *P*-value of < 0.05 was considered significant. All statistical analyses were performed using EZR software (Saitama Medical Center, Jichi Medical University), a graphical user interface for R (The R Foundation for Statistical Computing, version 2.13.0). More precisely, it is a modified version of R commander (version 1.6-3), which includes statistical functions frequently used in biostatistics.

## Results

### Patients

A total of 511 questionnaires were returned, of which 60 were excluded owing to missing items. Overall, 451 patients completed the survey. Two participants had come into close contact with confirmed cases of COVID-19, and one of these had undergone isolation; however, no cases of COVID-19 were enrolled in the study.

### Disease-Related Variables

Regarding specific diagnosis and disease activity before lockdown, 241 patients had UC (remission, 213 [88.4%]; mild activity, 14 [5.8%]; moderate activity, 11 [4.6%]; severe activity, 3 [1.2%]) and 210 patients had CD (remission, 123 [58.6%]; mild activity, 46 [21.9%]; moderate activity, 39 [18.6%]; severe activity, 2 [1.0%]). The median age at enrollment was 50 years (IQR 39–63) for both patients with UC and CD. The median age at diagnosis was 31 years (IQR 24–42) in patients with UC and 25 years (IQR 19–33) in patients with CD. The median disease duration was 13 years for both patients with UC (IQR 7–23) and CD (IQR 6–24). The detailed characteristics of the patients are shown in Table 1.

**Table 1:** Demographic data and disease-related variables of participants.

		**Ulcerative colitis**	**Crohn's disease**
Demographics	Number of patients	241	210
	Sex (male/female)	129/112	158/52
	Age at enrollment (years), median (IQR)	50 (39–63)	44 (34–50)
	Age at diagnosis (years), median (IQR)	31 (24–42)	25 (19–33)
	Disease duration (years), median (IQR)	13 (7–23)	13 (6–24)
	6-point Mayo score before the declaration of the state of emergency	0 (0–1)	
	6-point Mayo score during the state of emergency	0 (0–1)	
	PRO2 score before the declaration of the state of emergency		6 (0–11)
	PRO2 score during the state of emergency		9 (4–15)
Lifestyle during the state of emergency	Sleeping time (hours/day), mean (IQR)	6 (6–7)	6 (6–7)
	Working time (hours/week), median (IQR)	12 (0–40)	8 (0–8.75)
	Walking time (hours/day), median (IQR)	1 (0–1)	1 (0–1)
	Exercise time (minutes/week), median (IQR)	0 (0–120)	10 (0–40)
	Number of meals per day, median (IQR)	3 (3–3)	3 (2–3)
	Increased smoking	1 (0.4%)	14 (6.7%)
	Increased alcohol intake	29 (12.0%)	23 (11.0%)
	Deterioration of drug-adherence	3 (1.2%)	2 (1.0%)
Stress related to the state of emergency^†^	Stress due to childcare burden	2 (0.8%)	0 (0%)
	Stress due to COVID-19	14 (5.8%)	7 (3.3%)
	Stress due to family budget	10 (4.1%)	3 (1.4%)
	Stress due to inability to exercise	21 (8.7%)	10 (4.8%)
	Stress due to staying indoors	25 (10.4%)	18 (8.6%)
	Stress due to inflammatory bowel disease	7 (2.9%)	3 (1.4%)
	Stress due to worsening of diet and nutritional status	5 (2.1%)	2 (1.0%)
Medication	Mesalamine	214 (88.8%)	123 (58.6%)
	Enteral nutrition	0 (0%)	66 (31.4%)
	Corticosteroids	8 (3.3%)	8 (3.8%)
	Immunomodulators (azathioprine or 6-mercaptopurine)	64 (26.6%)	70 (33.3%)
	Anti-TNF therapy	31 (12.9%)	109 (51.9%)
	Ustekinumab	0 (0%)	26 (12.4%)
	Vedolizumab	11 (4.6%)	7 (3.3%)
	Tofacitinib	6 (2.5%)	not approved in Japan^*^
	Molecularly targeted therapies^**^	48 (19.9%)	141 (67.1%)

† *“Stress related to the state of emergency” was defined as newly emerging stress during the state of emergency*.

* *Tofacitinib is not approved for the treatment of Crohn's disease in Japan*.

** *“Molecularly targeted therapies” include anti-TNF therapy, ustekinumab, vedolizumab, and tofacitinib. IQR, interquartile range; COVID-19, coronavirus disease; PRO2, patient-reported outcome 2; TNF, tumor necrosis factor*.

### Impact of the Lockdown on Disease Activity, Lifestyle, and Psychological Stress

**Figure 1:**
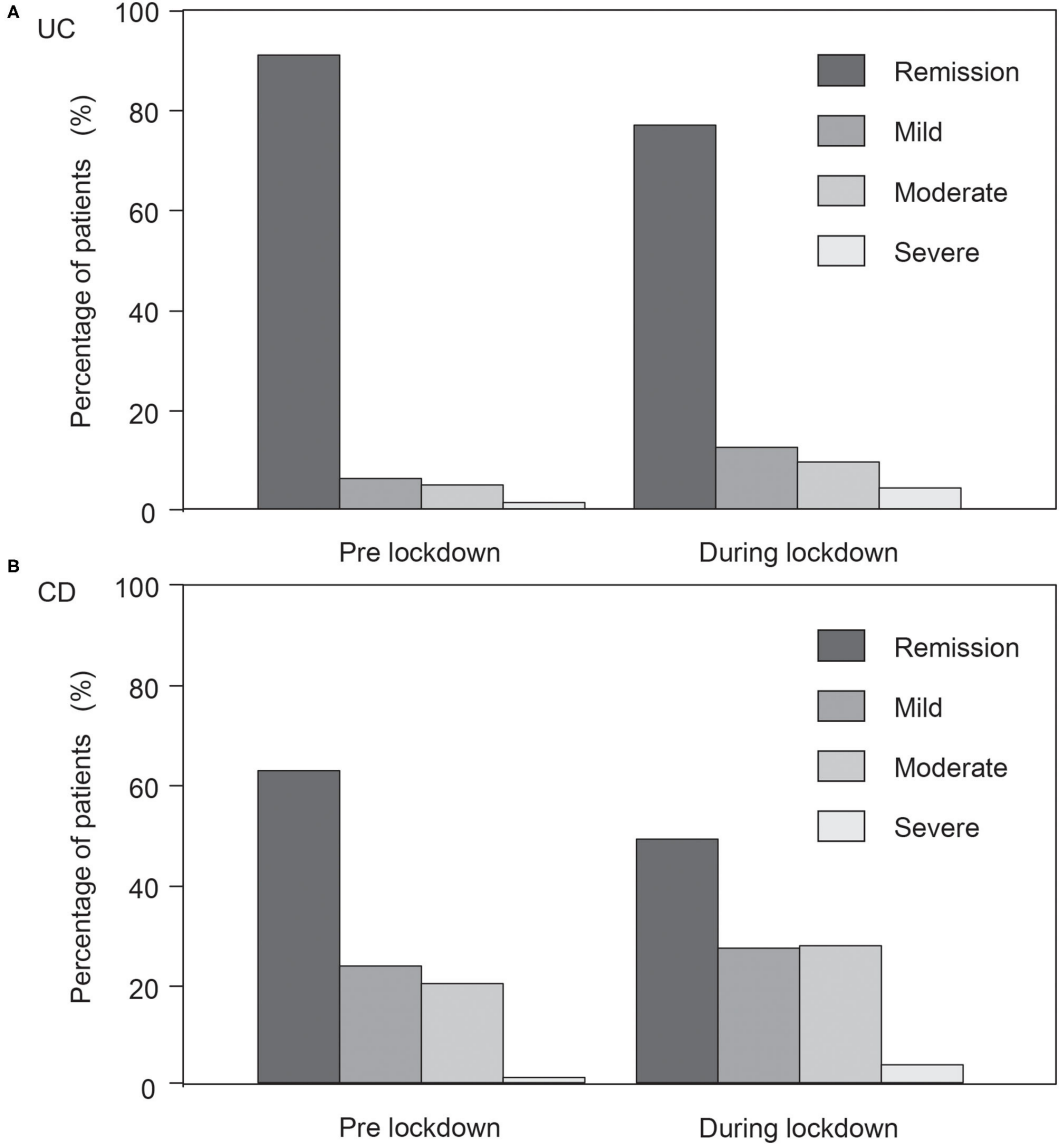
Comparison of disease activity before and during lockdown. **(A)** Ulcerative colitis (UC). **(B)** Crohn's disease (CD). Disease activity in UC changed as follows: remission, 213 (88.4%); mild activity, 14 (5.8%); moderate activity, 11 (4.6%); severe activity, 3 (1.2%) to remission, 180 (74.7%); mild activity, 29 (12.0%); moderate activity, 22 (9.1%); severe activity, 10 (4.1%). Disease activity in CD changed as follows: remission, 123 (58.6%); mild activity, 46 (21.9%); moderate activity, 39 (18.6%); severe activity, 2 (1.0%) to remission, 96 (45.7%); mild activity, 53 (25.2%); moderate activity, 54 (25.7%); severe activity, 2 (1.0%).

Within 2 months after the declaration of the state of emergency, gastrointestinal symptoms worsened in 34.0% and 46.2% of patients with UC and CD, respectively. Figure 1 shows a comparison of disease activity before and during lockdown. UC and CD activity after lockdown were as follows: UC (remission, 180 [74.7%]; mild activity, 29 [12.0%]; moderate activity, 22 [9.1%]; severe activity, 10 [4.1%]) and CD (remission, 96 [45.7%]; mild activity, 53 [25.2%]; moderate activity, 54 [25.7%]; severe activity, 2 [1.0%]). Additional treatment was only required for 14.6% and 12.4% of patients with exacerbated UC and CD, respectively. Among 213 patients with UC and 123 patients with CD who were in remission before lockdown, gastrointestinal symptoms worsened in 71 (33.3%) and 48 patients (39.0%), respectively. The rate of disease exacerbation did not significantly differ between all participants and those in remission for UC (*P* = 0.921) and CD (*P* = 0.21). In contrast, among 170 and 148 patients with UC and CD, respectively, who did not receive additional treatment due to disease exacerbation within 1 year before the state of emergency, gastrointestinal symptoms worsened in 54 (31.8%) and 63 patients (42.6%), respectively. The rate of disease exacerbation did not significantly differ between all participants and those with UC (*P* = 0.671) or CD (*P* = 0.519) who did not undergo additional treatment due to disease exacerbation within 1 year before the state of emergency. Regarding smoking, alcohol intake, and drug adherence, an increase in smoking was seen in 1 (0.4%) and 14 (6.7%), alcohol intake in 29 (12.0%) and 23 (11.0%), and a deterioration of drug-adherence in 3 (1.2%) and 2 (1.0%), UC and CD patients, respectively. Regarding psychological stress, a high percentage of people felt stress due to being forced to stay indoors or the inability to exercise, whereas the proportion of people with stress due to IBD or nutrition was not significantly high (Table 1).

### Risk Factors for Exacerbation

Table 2 shows a comparison of patient data. Patients with exacerbated UC (within 2 months after the declaration of the state of emergency) tended to be younger and had less sleep and more stress due to COVID-19 than patients with non-exacerbated UC. Multivariate logistic regression analysis identified age (odds ratio, OR 0.98, 95% CI 0.96–0.99; *P* < 0.05), sleep hours (OR, 0.74; 95% CI, 0.57–0.97; *P* < 0.05), and increased stress due to the COVID-19 pandemic (OR, 6.06; 95% CI, 1.79–20.50; *P* < 0.01) as independent risk factors associated with UC exacerbation (Table 3). Regarding patients with CD, those with exacerbations were lower age at enrollment, lower age at CD onset, and had active disease before the state of emergency than patients with non-exacerbated CD. However, multivariate analysis identified age (OR, 0.97; 95% CI, 0.95–0.99; *P* < 0.01) and active disease before the state of emergency (OR, 2.20; 95% CI, 1.23–3.95; *P* < 0.01) as independent factors associated with CD exacerbation (Table 3). Alcohol increase, smoking increase, and drug adherence change were not identified as independent risk factors for exacerbation.

**Table 2:** Descriptive comparison of participants with and without exacerbation.

		**Patients with ulcerative colitis**			**Patients with Crohn's disease**		
		**Non-exacerbated**	**Exacerbated**	***P*** **-value**	**Non-exacerbated**	**Exacerbated**	***P*** **-value**
Demographics	Number of patients	159	82		113	97	
	Sex (male/female)	82/77	47/35	0.416	85/28	73/24	1
	Age at enrolment (years), median (IQR)	51 (39–66)	46.5 (39–56.5)	0.051	46 (35–54)	42 (31–48)	0.014
	Age at diagnosis (years), median (IQR)	32 (24.5–43)	30 (22–41.75)	0.169	26 (20–38)	24 (18–28)	0.013
	Disease duration (years), median (IQR)	13 (7–23)	13.5 (5–22.25)	0.533	13 (5–25)	15 (7–22)	0.788
	6-point Mayo score before the declaration of the state of emergency	0 (0–1)	0 (0–1)	0.221			
	PRO2 score before the declaration of the state of emergency				5 (0–10)	8 (2–13)	0.025
Lifestyle during the state of emergency	Sleeping time (hours/day), mean (IQR)	6 (6–7)	6 (6–7)	0.073	6 (6–7)	7 (6–7)	0.763
	Working time (hours/week), median (IQR)	10 (0–40)	16 (0–40)	0.171	8 (0–9)	8 (0–8)	0.901
	Walking time (hours/day), median (IQR)	1 (0–1)	1 (0–1)	0.295	1 (1–1)	1 (0–1)	0.491
	Exercise time (minutes/week), median (IQR)	0 (0–120)	0 (0–120)	0.917	10 (0–60)	15 (0–30)	0.819
	Number of meals per day, median (IQR)	3 (3–3)	3 (3–3)	0.493	3 (2–3)	3 (2–3)	0.593
	Increased smoking	1 (0.6%)	0 (0.0%)	1	8 (7.1%)	6 (6.2%)	1
	Increased alcohol intake	17 (10.7%)	12 (14.6%)	0.406	12 (10.6%)	11 (11.3%)	1
	Deterioration of drug-adherence	1 (0.6%)	2 (2.4%)	0.268	1 (1.0%)	1 (1.1%)	1
Stress related to the state of emergency^†^	Stress due to childcare burden	1 (0.6%)	1 (1.2%)	1	0 (0%)	0 (0%)	NA
	Stress due to COVID-19	4 (2.5%)	10 (12.2%)	0.006	2 (1.8%)	5 (5.2%)	0.253
	Stress due to family budget	7 (4.4%)	3 (3.7%)	1	1 (0.9%)	2 (2.1%)	0.597
	Stress due to inability to exercise	12(7.5%)	9 (11%)	0.47	4 (3.5%)	6 (6.2%)	0.519
	Stress due to staying indoors	15(9.4%)	10 (12.2%)	1	11 (9.7%)	7 (7.2%)	0.624
	Stress due to inflammatory bowel disease	3 (1.9%)	4 (4.9%)	0.51	2 (1.8%)	1 (1.0%)	1
	Stress due to worsening of diet and nutritional status	3 (1.9%)	2 (2.4%)	0.55	0 (0%)	2 (2.1%)	0.212
Medication	Mesalamine	142 (89.3%)	72 (87.8%)	0.83	67 (59.3%)	56 (57.7%)	0.888
	Enteral nutrition	0 (0%)	0 (0%)	NA	36 (31.9%)	30 (30.9%)	1
	Corticosteroids	5 (3.1%)	3 (3.7%)	1	3 (2.7%)	5 (5.2%)	0.475
	Immunomodulators (azathioprine or 6-mercaptopurine)	47 (29.6%)	17 (20.7%)	0.167	35 (31%)	35 (36.1%)	0.465
	Anti-TNF therapy	17 (10.7%)	14 (17.1%)	0.222	55 (48.7%)	54 (55.7%)	0.335
	Ustekinumab	0 (0%)	0 (0%)	NA	13 (11.5%)	13 (13.4%)	0.681
	Vedolizumab	8 (5%)	3 (3.7%)	0.754	2 (1.8%)	5 (5.2%)	0.253
	Tofacitinib	3 (1.9%)	3 (3.7%)	0.404	Not approved in Japan		
	Molecularly targeted therapies^**^	28 (17.6%)	20 (24.4%)	0.235	70 (61.9%)	71 (73.2%)	0.105

† “Stress related to the state of emergency” was defined as newly emerging stress during the state of emergency. ^*^Tofacitinib is not approved for the treatment of Crohn's disease in Japan.

** *“Molecularly targeted therapies” include anti-TNF therapy, ustekinumab, vedolizumab, and tofacitinib. COVID-19, coronavirus disease; IQR, interquartile range; PRO2, patient-reported outcome 2; TNF, tumor necrosis factor*.

**Table 3:** Logistic regression analyses of factors associated with exacerbation.

**Patients with UC**	**Univariate OR (95% CI)**	***P*** **-value**	**Multivariate OR (95% CI)^†^**	***P*** **-value**
Age at enrollment	0.98 (0.96–0.99)	0.036	0.98 (0.96–0.99)	<0.05
Age at onset	0.98 (0.98–1.00)	0.106		
Sleep hours	0.81 (0.63–1.03)	0.086	0.74 (0.57–0.97)	<0.05
Stress due to the COVID-19 pandemic				
No	Ref		Ref	
Yes	5.38 (1.63–17.70)	<0.01	6.06 (1.79–20.50)	<0.01
Disease activity				
Remission	Ref		Ref	
Active	1.29 (0.58–2.91)	0.53	1.27 (0.53–3.04)	0.59
Smoking habit				
Decrease / No change	Ref		Ref	
Increase	9.10e-7 (0–inf)	0.99	7.28e-7 (0–inf)	0.98
Alcohol intake				
Decrease / No change	Ref		Ref	
Increase	1.43 (0.65–3.16)	0.38	1.69 (0.74–3.85)	0.22
Drug adherence				
Improvement / No change	Ref		Ref	
Deterioration	3.95 (0.35–44.20)	0.27	3.02 (0.25–36.5)	0.39
**Patients with CD**	**Univariate OR (95% CI)**	***P*** **-value**	**Multivariate OR (95% CI)**	***P*** **-value**
Age at enrollment	0.97 (0.95–0.99)	<0.05	0.97 (0.94–0.99)	<0.01
Age at onset	0.97 (0.94–0.99)	<0.01		
Sleep hours	1.00 (0.77–1.29)	0.99	0.97 (0.74–1.27)	0.81
Stress due to the COVID-19 pandemic				
No	Ref		Ref	
Yes	3.02 (0.57–15.90)	0.19	3.69 (0.64–21.40)	0.15
Disease activity				
Remission	Ref		Ref	
Active	2.01 (1.15–3.52)	<0.05	2.20 (1.23–3.95)	<0.01
Smoking habit				
Decrease / No change	Ref		Ref	
Increase	0.86 (0.29–2.59)	0.8	0.88 (0.26–3.03)	0.84
Alcohol intake				
Decrease / No change	Ref		Ref	
Increase	1.08 (0.45–2.56)	0.87	0.89 (0.35–2.30)	0.82
Drug adherence				
Improvement / No change	Ref		Ref	
Deterioration	1.17 (0.07–18.90)	0.91	1.21 (0.07–21.80)	0.90

*Age at enrollment, age at onset and sleep hours were considered as continuous variables*.

† *Adjusted for factors, including age at enrollment, sex, short sleep, stress due to the COVID-19 pandemic, increased smoking, increased alcohol intake, drug adherence deterioration, and active disease. COVID-19, coronavirus disease; PRO2, patient-reported outcome 2; UC, ulcerative colitis; CD, Crohn's disease; CI, confidence interval; OR, odds ratio*.

## Discussion

Our results suggest that changes in daily life and stress status due to the pandemic and lockdown measures were associated with worsening IBD symptoms, especially in young patients. Possible explanations for these findings could be as follows. First, according to a recent report, the negative impact of lockdown measures on daily life may be more prevalent in younger people than in older people (4). The impact of age at IBD onset on the natural history, severity, and surgical rates have been reported to be higher in patients with elderly-onset UC than in patients with non-elderly-onset UC (17–20), whereas the rates of disease progression have been shown to be lower in patients with elderly-onset CD than in patients with non-elderly-onset CD (21, 22). In the current study, although both patients with UC and CD with worsening IBD symptoms were younger than those without worsening symptoms at enrollment, only patients with CD with worsening symptoms were younger at CD onset than those without worsening symptoms. These results indicate that patients with UC might experience episodes of exacerbations due to the impact of the lockdown, but not natural history, in contrast to patients with CD.

Second, sleep disturbances are commonly seen in patients with active IBD (23, 24) and are associated with the onset of UC (24). Ananthakrishnan et al. reported that sleep disturbance was associated with an increased risk of CD but not UC exacerbation (25). In the current study, multivariate logistic regression analysis identified fewer sleep hours as an independent risk factor associated with UC but not with CD exacerbation. This discrepancy may occur owing to the quality of sleep. Only sleep time could be evaluated as a sleep factor, as the questionnaire used in this study did not include questions associated with sleep disturbance or use of medications that could estimate the quality of sleep. Therefore, further studies will be required to explain this discrepancy.

Finally, stress resulting from the fear of contracting a potentially lethal disease that affects mostly immunosuppressed individuals might aggravate IBD symptoms, though an inverse relation cannot be excluded (IBD exacerbation could cause psychological stress). Several studies have reported the psychological impact of the pandemic on the general population and demonstrated an increase in the level of anxiety during the pandemic, and patients with IBD are more likely to develop anxiety disorders (26–28).

In this study, no case of COVID-19 was registered; however, this does not mean patients with IBD were less likely to contract COVID-19. This is probably owing to the small sample size and low infection rate of COVID-19 during this period in Japan. Indeed, IBD *per se* does not increase the risk of developing COVID-19 (29), and patients with IBD receiving immunomodulators, biological agents, or JAK inhibitors do not have an increased risk of contracting SARS-CoV-2 infection or developing a more severe course of infection (30). Only corticosteroid use was reported to be associated with severe COVID-19 among patients with IBD (31). However, elderly patients or those with comorbidities have a poorer clinical outcome after contracting COVID-19 (30, 32, 33). Based on this evidence and the results of our study, older patients or those with current use of corticosteroid treatments need thorough observation and early intervention to prevent the potential development of severe COVID-19. In addition, younger patients should be careful to prevent exacerbations of IBD during lockdown because they are likely to worsen. Additionally, the results of our study suggested that patients in remission or those who did not require additional treatment within 1 year before the state of emergency had a similar risk of disease exacerbation during the state of emergency. The multivariate logistic regression analysis that included scores for gastrointestinal symptoms also supported this finding.

This study has some limitations, including its single-center nature and relatively small cohort, which could be prone to bias in data selection and analysis. Moreover, the results of our study should have been compared with the rate and factors for gastrointestinal disease exacerbation before the COVID-19 pandemic occurred; however, this comparison could not be performed because of the lack of relevant pre-COVID-19 pandemic data for these diseases. Additionally, we were unable to evaluate objective factors (e.g., laboratory examinations, endoscopic activities, disease locations) because anonymity needed to be maintained in the questionnaire. Patients who experienced disease exacerbation may have had a functional disorder, but it was difficult to assess the influence of such based only on subjective factors. Only 14.6 and 12.4% of patients with exacerbated UC and CD, respectively, required additional treatment, some of which may have had a functional disorder or experienced mild exacerbation of the disease. Disease exacerbation in this study was defined as an increase of 1 point or more in the 6-point Mayo or PRO2 scores. However, since disease exacerbation was not evaluated objectively, it could not be quantified. Further, change in the line of treatments for IBD flare-up during the COVID-19 pandemic should have been evaluated, as alterations could have been made throughout this period that may have affected our results. For example, the European Crohn's and Colitis Organization position statement recommended the use of subcutaneous drugs for IBD flare-ups to minimize hospital visits (34). However, we could not evaluate the details of the additional treatment for the exacerbation because the questionnaire did not include these items. Further, we used simple questions rather than validated ones for psychological factors to reduce the burden on respondents and increase the response rate in consideration of the large number of questions.

Another possible limitation of this study was possible selection bias. Since patients visiting the clinic are likely to have more symptoms (or less), the results may be biased and not generalizable to all patients with IBD. In addition, this study was conducted in a single-tertiary center, which may suggest the patients have more complicated disease. However, in Japan, almost all patients with IBD visit the clinic regularly even if they have no symptoms. In addition, it is difficult to include patients with IBD without clinical visits. Although our hospital is a tertiary medical institution, it also provides regular follow-up for patients with remission or mild IBD. Therefore, this may only moderately limit the generalizability of the findings.

Furthermore, as the questionnaire was completed based on memory recall, a response bias could have influenced the answers of the study participants. This may be owing to fatigue from answering many questions or difficulty in remembering gastrointestinal symptoms or lifestyles before the state of emergency; thus, 60 of 511 participants (11.7%) could not complete the questionnaire. As this was a retrospective study, a possibility of reverse causality may have occurred. It is possible that patients were having more symptoms from their disease due to exacerbation, which in turn led to poor sleep and increasing stress. Therefore, further large prospective studies are needed to confirm the impact of a lockdown on patients with IBD.

This study is the first to provide data on the association between IBD activity and lifestyle changes/psychological stress due to the state of emergency during the COVID-19 pandemic. Our finding suggests that improving the quality of sleep and preventing psychological stress may be significant factors in improving IBD management during a pandemic, especially among young patients.

## Data Availability Statement

The raw data supporting the conclusions of this article will be made available by the authors, without undue reservation.

## Ethics Statement

The studies involving human participants were reviewed and approved by the Ethics Committee of the Osaka City University Graduate School of Medicine. Written informed consent from the participants' legal guardian/next of kin was not required to participate in this study in accordance with the national legislation and the institutional requirements.

## Author Contributions

SH conceived the study and supervised the overall study. YNi and SH wrote the manuscript. Data collection and analysis were performed by YNi, SH, KF, SI, NK, and SO. All authors contributed to the article and approved the submitted version.

## Conflict of Interest

The authors declare that the research was conducted in the absence of any commercial or financial relationships that could be construed as a potential conflict of interest.

## Publisher's Note

All claims expressed in this article are solely those of the authors and do not necessarily represent those of their affiliated organizations, or those of the publisher, the editors and the reviewers. Any product that may be evaluated in this article, or claim that may be made by its manufacturer, is not guaranteed or endorsed by the publisher.

## Abbreviations

COVID-19: coronavirus disease
CD: Crohn's disease
IBD: inflammatory bowel disease
OR: odds ratio
PRO2: patient-reported outcome 2
SARS-CoV-2: severe acute respiratory syndrome coronavirus 2
UC: ulcerative colitis.
